# Clinical Outcomes of Micropulse Transscleral Cyclophotocoagulation in Patients with a History of Keratoplasty

**DOI:** 10.1155/2020/6147248

**Published:** 2020-07-09

**Authors:** Jun Hui Lee, Vivian Vu, Gabriel Lazcano-Gomez, Katherine Han, Pukkapol Suvannachart, Jennifer Rose-Nussbaumer, Julie Schallhorn, David Hwang, Ying Han

**Affiliations:** ^1^Yale School of Medicine, New Haven, CT, USA; ^2^University of California, San Francisco, School of Medicine, San Francisco, CA, USA; ^3^Department of Ophthalmology, University of California, San Francisco, School of Medicine, San Francisco, CA, USA

## Abstract

**Purpose:**

To examine the surgical outcomes and graft conditions in patients receiving micropulse transscleral cyclophotocoagulation (MP-TSCPC) to treat post-keratoplasty ocular hypertension.

**Methods:**

This retrospective observational study included 30 eyes of 28 consecutive glaucoma patients with a history of penetrating keratoplasty (PKP) or Descemet's stripping automated endothelial keratoplasty (DSAEK) who underwent MP-TSCPC at the University of California, San Francisco from 09/2015 to 08/2018. Using the Wilcoxon signed-rank test, we compared preoperative and postoperative intraocular pressure (IOP), number of glaucoma medications, visual acuity, and central corneal thickness at 1, 3, 6, and 12 months. Postoperative complications, additional surgeries, and graft failures were also recorded at these follow-up times. Linear regression model was used to study whether PKP vs. DSAEK affects the effectiveness of MP-TSCPC.

**Results:**

Thirty eyes from 28 patients were followed for 12 months. IOP was significantly decreased from preop at all follow-up points (*P* < 0.001). There was no significant change in the number of glaucoma drops, visual acuity, or CCT. At 12 months, 21 of the 30 eyes met the definition of success, and only one underwent repeat PKP due to graft rejection. The type of corneal transplant was not a significant factor for IOP reduction at the last follow-up.

**Conclusions:**

MP-TSCPC achieved desirable IOP control and success rates for postkeratoplasty patients while resulting in minimal complications and graft failure. It appears to be a safe and effective procedure in patients who received corneal transplant with one-year follow-up.

## 1. Introduction 

The progression of glaucoma following corneal transplantation is a well-known source of ocular morbidity [1]. The common causes of postkeratoplasty intraocular pressure (IOP) elevation are synechial angle closure [2] and the need for chronic corticosteroid use [3]. Other factors considered include postoperative inflammation and trabecular mesh dysfunction [4]. Uncontrolled IOP leads to optic nerve damage and increased rate of corneal endothelial cell loss, subsequently causing permanent visual loss in postkeratoplasty patients [5, 6]. When pharmacotherapy is no longer effective, procedural interventions may be required.

However, to date, there is no ideal intervention for these patients. Laser trabeculoplasty in postkeratoplasty eyes is not commonly used due to limited view of the angle structure through the cloudy peripheral cornea [7]. Glaucoma incisional surgery, including trabeculectomy or glaucoma drainage device (GDD), provides excellent IOP control but may lead to the failure of the graft due to mechanical disturbance of endothelial cells, postoperative tube-endothelium touch, and/or the toxicity of antimetabolite adjunct therapy [8–10]. Previous studies have shown that incisional surgeries are associated with a shorter time to graft rejection, a greater likelihood of multiple rejection episodes, and a greater chance of graft failure when compared with nonglaucomatous or medically treated eyes [5, 11, 12].

Traditional cyclodestructive laser procedures, such as diode laser transscleral cyclophotocoagulation (TSCPC), can be useful tools in controlling the IOP after keratoplasty. However, TSCPC has been associated with higher incidence of graft failure, hypotony, and visual loss [13]. Furthermore, the risk of vision-threatening complications such as phthisis bulbi has largely relegated this procedure to end-stage glaucoma patients [14].

There is a need for a treatment option that can control the IOP without disturbing the corneal graft. Micropulse transscleral cyclophotocoagulation (MP-TSCPC) is a new treatment for glaucoma that updates traditional TSCPC with micropulse technology. Previous studies showed that it offers satisfactory IOP control with less complications compared to the continuous wave TSCPC [15–18]. Furthermore, it does not require an incision or tube placement and does not risk disrupting the intraocular flow that may damage the corneal endothelium. Therefore, we hypothesized that MP-TSCPC may be a new method to reduce the IOP while sparing the graft in ocular hypertension patients with keratoplasty.

In this study, we conducted a retrospective study on consecutive patients with a history of penetrating keratoplasty (PKP) or Descemet's stripping automated endothelial keratoplasty (DSAEK) who received MP-TSCPC therapy for IOP control. The surgical outcome and graft thickness were examined over 1-year follow-up period.

## 2. Materials and Methods

This was a retrospective observational study involving consecutive glaucoma patients with a history of PKP or DSAEK who underwent MP-TSCPC at the University of California, San Francisco (UCSF) Glaucoma Clinic from September 2015 to August 2018. All patients who needed MP-TSCPC after corneal transplant during this period were included in the study. This study was approved by the UCSF Committee on Human Research and upheld the tenets of Declaration of Helsinki.

The MicroPulse P3 treatment, which belongs to the CYCLO G6 Glaucoma Laser System (IRIDEX, Inc., Mountain View, CA), was used in all subjects. The patients received either a retrobulbar block or general anesthesia. The G6 probe was placed at the limbus with the probe perpendicular to the surface of the globe. Laser settings were 2000 mW with a duty cycle of 31.33%. The laser was applied throughout 180° over 160 seconds. The same procedure was then repeated for the other hemifield.

All patients received intensive steroid treatment during MP-TSCPC, including IV Solu-Medral 500 mg. Postoperative prednisolone ophthalmic solution (1%) was used initially every 1-2 hours and gradually tapered to baseline over a 3-month period.

Along with demographic and clinical characteristics, the following parameters were recorded for each patient: preoperative and postoperative IOP by pneumatonometry, number of glaucoma medications, visual acuity, central corneal thickness (CCT), and postoperative complications at 1 month, 3 months, 6 months, and 12 months of follow-up. Additional glaucoma surgeries were recorded for patients who failed to respond to MP-TSCPC treatment. The rate of failure of the graft is recorded at 12-month follow-up.

### 2.1. Definition of Success

Success of MP-TSCPC was defined as (1) 5 mmHg ≤ IOP ≤ 21 mmHg and reduced ≥20% from baseline at the last follow-up; (2) no use of oral carbonic anhydrase inhibitors; (3) no loss of light perception vision; and (4) no reoperation for glaucoma within the 12-month follow-up period.

### 2.2. Statistical Analysis

Preoperative and postoperative IOP, number of glaucoma medications, visual acuity, and CCT were compared using the Wilcoxon signed-rank test. Linear regression model was used to study whether PKP vs. DSAEK influences the effectiveness of MP-TSCPC. Statistical calculations were performed with SPSS (IBM SPSS Statistics 22.0) with *P* < 0.05, before Bonferroni correction, denoting the statistical significance of differences.

## 3. Results

A total of 30 eyes from 28 patients were included in the study. Every eye had 12-month follow-up except for one eye which was lost to follow-up after undergoing additional surgery at POM6. Of total, 16 eyes were post-PKP and 14 were post-DSAEK. The average age was 65.9 years, and 43.3% were males. Of total, 73.3% had a history of cataract surgery, 46.3% had a history of previous glaucoma surgery, and 50% had a history of repeat corneal transplant. The median time between the last corneal transplant and the MP-TSCPC treatment was 15.2 months. Preoperatively, 29% of the patients were using oral CAI for IOP control (Table 1).

### 3.1. Outcome of MP-TSCPC

**Figure 1:**
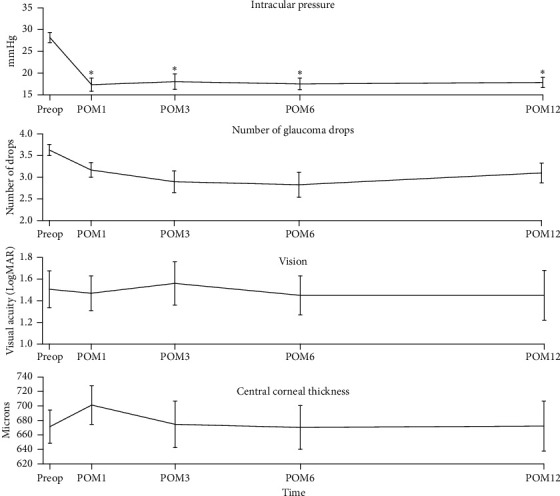
Clinical outcomes following MP-TSCPC in patients with a history of corneal transplant.

The mean values for IOP, number of glaucoma medications, visual acuity in LogMAR, and CCT for preop, POM 1, 3, 6, and 12 months are listed in Table 2. For the eyes that received additional glaucoma surgery, the IOP measurement and the number of drops at the last follow-up period before the surgery were carried over to subsequent follow-up periods for analysis. IOP was significantly decreased from preop at all follow-up points. The number of glaucoma medications was not significantly reduced at any point when Bonferroni-corrected *P* value was applied. There was no change in visual acuity or CCT over the 12-month follow-up (Figure 1).

At the last follow-up, 70% (21 out of 30 eyes) of the eyes met the definition of success for MP-TSCPC. Five eyes underwent other glaucoma surgeries (1 ECP and 4 filtering surgery) for IOP control. One of those eyes also required oral CAI at POM12. Other 4 eyes failed due to inadequate IOP reduction.

The most common early (<1 month) postprocedure complication was AC inflammation, occurring in 40% of the eyes. However, the inflammation did not last longer than 3 months. One eye had corneal epithelial defect and one eye had a choroidal effusion, which were both resolved before the 3-month visit. Late complications included hypotony (1 eye) and macular edema (1 eye) (Table 3).

### 3.2. Graft Outcomes

Over the 12-month follow-up, there were no significant changes in CCT; although CCT at POM1 was higher than baseline, the difference did not reach statistical significance (*P*=0.56). Over the 12-month follow-up, one eye (3.3%) underwent repeat PKP at POM2 due to graft rejection.

**Figure 2:**
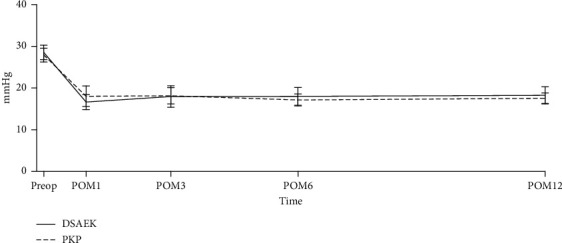
Intraocular pressures following MP-TSCPC in patients with history PKP vs. DSAEK.

There were 16 patients who were post-PKP and 14 who were post-DSAEK in our study. To examine whether MP-TSCPC has different IOP-lowering effect with different types of postcorneal transplant patients, we graphed the IOP outcome for both groups (Figure 2). We also performed linear regression on the percentage of IOP reduction at the last follow-up using whether the patient was post-PKP or post-DSAEK and age as independent variables (Table 4). When controlling for age, the type of corneal transplant did not affect the IOP-lowering effect of MP-TSCPC. In addition, there were no differences in the rate of any of the complications observed in this study between post-PKP and post-DSAEK patients.

## 4. Discussion

In this retrospective study, we analyzed the clinical outcomes of MP-TSCPC in postkeratoplasty glaucoma patients. To our knowledge, this is the first study to investigate the effect of MP-TSCPC in this population. At the 12-month follow-up, the average IOP was significantly decreased at all follow-up points. Although there was no significant change in the number of topical eye drops, only one patient was on oral CAI, while 8 patients took oral CAI before the treatment. There was no reduction in visual acuity or increase in CCT. Only one eye (3.3%) required repeat PKP for the sole graft failure among this group. In addition, the type of corneal transplant was not a factor associated with IOP reduction or postoperative complication rate after MP-TSCPC.

Overall, we found that IOP reduction after MP-TSCPC for postkeratoplasty glaucoma patients is comparable to that reported in previous studies which focused mainly on patients with primary open-angle glaucoma [15–17]. For example, Williams et al. reported 75% success rate at 12 months with average IOP reduction of 51% [19]. Yelenskiy et al. similarly reported 71% success rate with 27% decrease in mean IOP at 12 months [20]. These results are consistent with those of the present study with mean IOP reduction of 36.6% at POM12 follow-up and success rate of 70%.

Conventional diode TSCPC has been used to treat elevated IOP in patients with postkeratoplasty glaucoma [21–23]. While studies on the treatment of postkeratoplasty glaucoma with diode TSCPC overall report adequate control of IOP in most patients (range 64–85%), a considerable portion of those patients (average 43.5%) reported significant decrease in the Snellen visual acuity. Moreover, an average of 32.8% developed graft failure. Other complications included anterior uveitis, epithelial defects, severe pain, phthisis bulbi, hyphema, hypopyon, and sympathetic ophthalmia [13]. It appears that the use of MP-TSCPC is comparable to the conventional TSCPC in terms of rate of success in IOP control, yet much less likely to induce graft failure or other complications that may lead to repeat PKP/DSAEK. The fact that the CCT did not increase at any of our follow-up points also supports that MP-TSCPC has relatively mild effect on the cornea graft [24, 25].

Endocyclophotocoagulation (ECP) is another cyclodestructive procedure that may be used in patients with postkeratoplasty glaucoma. A retrospective study by Chen et al. reported that 16 patients with pre-existing corneal transplant underwent ECP and only 1 (6%) eye showed signs of graft rejection episode during mean follow-up time of 15.4 ± 6.1 months. A more recent study by Huang et al. compared ECP and diode conventional TSCPC in patients with a history of postkeratopathy [26]. In this retrospective study, ECP proved more effective than TCP in controlling IOP in postkeratopathy glaucoma patients at 6 months. Furthermore, the patients in the ECP group showed less endothelial cell loss. While ECP is effective in controlling IOP for patients with keratoplasty, it requires incision. MP-TSCPC may achieve similar outcome but does not need any incision. A comparative study between the two studies is warranted in the future.

Trabeculectomy and GDDs have also been used to treat patients with postkeratoplasty glaucoma with excellent IOP results [13]. A retrospective study on the surgical outcomes for patients with intractable glaucoma after PK reported 76.5% and 80% success rates following trabeculectomy with mitomycin C and GDD groups, respectively [27]. Other studies focusing on GDD reported the rate of adequate IOP control at 1 year to be between 74 and 96% [28, 29]. However, graft condition is a concern with trabeculectomy and tube shunt surgery. Graft survival in the setting of postkeratopathy and tube shunt ranges from 58.5% to 96% at 1 year [6]. Retrospective analysis on the risk factors of corneal graft failure showed that GDD presence is a significant predictor [29–32]. Almousa et al. also reported that 13% of eyes following AGV placement developed corneal graft opacity at 1 year [33]. Out of 13 patients whose graft was clear before trabeculectomy, 2 (15.4%) patients had their transplant develop opacity at 22 and 44 months [13]. The use of antimetabolite such as MMC and the risk of tube-endothelium touch have been described as possible causes of corneal graft failure. Alternatively, MP-TSCPC may afford a comparable rate of success in controlling the IOP while not requiring intraocular hardware nor adjunct antimetabolite therapy.

There are several limitations to this study. First, this is a retrospective study with limited sample size. In order to make a more generalized statement on the effect of MP-TSCPC, a larger prospective investigation in patients with a history of corneal transplant is warranted. Additionally, in terms of postkeratoplasty measures, we only examined CCT and incidence of graft failure. More detailed parameters, such as endothelial cell count, may provide more information on the role of MP-TSCPC in postkeratoplasty glaucoma management. Lastly, as one-year follow-up may not be long enough to evaluate the graft failure, [34], a future study with longer follow-up period is warranted.

In conclusion, MP-TSCPC appears to be a safe and effective procedure in postkeratoplasty glaucoma patients with minimal complications with one-year follow-up. The efficacy of MP-TSCPC in this population appears comparable to that in POAG patients. Pending future investigation with greater generalizability, MP-TSCPC may be considered a preferable treatment option for patients with postkeratoplasty glaucoma.

## Figures and Tables

**Table 1:** Demographic and clinical characteristics.

Total eyes/patients		30/28
Age (±SD)		65.9 (±20.2)
Male		43.3%
Race	Caucasian	43.4%
	Hispanic	26.7%
	Asian	23.3%
	African American	6.7%
History of cataract surgery		73.3%
Previous glaucoma surgery	Trabeculectomy	7/23.3%
	Glaucoma tube	8/26.7%
	ECP	5/16.7%
	TSCPC	2/6.7%
	MP-TSCPC	1/3.3%
	None	16/53.3%

Type of corneal transplant	PK	53.30%
	DSAEK	46.70%
History of repeat corneal transplant before MP-TSCPC		50.0%
Time between corneal transplant and MP-TSCPC (median, months)		15.2
Number of pre-MP-TSCPC glaucoma drops		3.63 (±0.72)
Preop use of oral CAI		29.0%
Preop IOP		27.7 (±6.77)
Preop VA (LogMAR)		1.51 (±0.95)

**Table 2:** Summary of clinical outcomes following MP-TSCPC in patients with a history of corneal transplant.

Time	IOP (mmHg) (±std error)	*P*	#Drops (±std error)	*P*	LogMAR (±std error)	*P*	CCT (micron) (±std error)	*P*
Preop (*n* = 30)	28.2 ± 1.18		3.63 ± 0.13		1.51 ± 0.17		671.52 ± 22.91	
POM1 (*n* = 30)	17.36 ± 1.50	**<0.001**	3.17 ± 0.17	0.041	1.47 ± 0.16	0.87	701.27 ± 26.77	0.56
POM3 (*n* = 30)	18.05 ± 1.77	**<0.001**	2.90 ± 0.25	0.010	1.56 ± 0.20	0.92	674.61 ± 32.27	0.94
POM6 (*n* = 30)	17.53 ± 1.34	**<0.001**	2.83 ± 0.29	0.006	1.45 ± 0.18	0.54	670.37 ± 30.22	0.83
POM12 (*n* = 29)	17.88 ± 1.18	**<0.001**	3.10 ± 0.23	0.049	1.45 ± 0.23	0.99	672.21 ± 34.73	0.29

**Table 3:** Summary of complications after MP-TSCPC in postkeratoplasty glaucoma patients.

Type of complication	Number of eyes (%)
<1 month	
Corneal epithelial defect	1 (3.3%)
AC inflammation	12 (40%)
Choroidal effusion	1 (3.3%)
Hypotony, macular edema, and hyphema	0
>1 month	
Hypotony	1 (3.3%)
Macular edema	1 (3.3%)
Hyphema, corneal epithelial defect, and choroidal effusion	0

**Table 4:** Linear regression analysis on percent IOP reduction at the last follow-up.

	Coefficient	Standard error	*P*	95% CI	
DSAEK	4.58	9.08	0.62	−14.04	23.20
Age	−0.17	0.23	0.46	−0.64	0.30
Const	46.17	13.23	0.002	19.03	73.32

## Data Availability

The data used to support the findings of this study are available from the corresponding author upon request.
